# Designing microorganisms for heterologous biosynthesis of cannabinoids

**DOI:** 10.1093/femsyr/fox037

**Published:** 2017-06-04

**Authors:** Ângela Carvalho, Esben Halkjær Hansen, Oliver Kayser, Simon Carlsen, Felix Stehle

**Affiliations:** 1 Evolva Biotech A/S, Lersø Parkallé 42-44, 2100, Copenhagen, Denmark; 2 Laboratory of Technical Biochemistry, Department of Biochemical and Chemical Engineering, TU Dortmund University, Emil-Figge-Str. 66, 44227 Dortmund, Germany

**Keywords:** *Cannabis sativa*, cannabinoids, synthetic biology, biotechnology, *Saccharomyces cerevisiae*

## Abstract

During the last decade, the use of medical *Cannabis* has expanded globally and legislation is getting more liberal in many countries, facilitating the research on cannabinoids. The unique interaction of cannabinoids with the human endocannabinoid system makes these compounds an interesting target to be studied as therapeutic agents for the treatment of several medical conditions. However, currently there are important limitations in the study, production and use of cannabinoids as pharmaceutical drugs. Besides the main constituent tetrahydrocannabinolic acid, the structurally related compound cannabidiol is of high interest as drug candidate. From the more than 100 known cannabinoids reported, most can only be extracted in very low amounts and their pharmacological profile has not been determined. Today, cannabinoids are isolated from the strictly regulated *Cannabis* plant, and the supply of compounds with sufficient quality is a major problem. Biotechnological production could be an attractive alternative mode of production. Herein, we explore the potential use of synthetic biology as an alternative strategy for synthesis of cannabinoids in heterologous hosts. We summarize the current knowledge surrounding cannabinoids biosynthesis and present a comprehensive description of the key steps of the genuine and artificial pathway, systems biotechnology needs and platform optimization.

## Teaser

In this review, the authors explore the use of synthetic biology as an alternative approach for the synthesis of pharmaceutical cannabinoids in a heterologous host organism.

## INTRODUCTION

Cannabinoids enclose a group of more than 100 chemical compounds (Ahmed *et al.*^2008^; ElSohly and Slade 2005; Radwan *et al.*^2008^) mainly found in the plant *Cannabis sativa* L., native from Central Asia (de Barge 1860; de Candolle 1886). *Cannabis* belongs to the family *Cannabaceae* that comprise only 11 genera, including *Cannabis*, *Humulus* (hop) and *Celtis* (hackberries). *C. sativa* is an annual plant with a dioecious breeding system (i.e. male and female flowers are found on separate plants), although monoecious plant also exist as result of special breeding (Clarke 1981; Raman 1998). The tendency in literature is to use the designation *C. sativa* L. to all species or varieties of the genera *Cannabis*. Despite this fact the number of species is not consensual, with some authors proposing a monotypic genus while others argue the existence of four (*sativa*, *indica*, *ruderalis* and *afghanica*) or even seven (*ruderalis*, *sativa* ssp. *sativa*, *sativa* ssp. *spontanea*, *indica* ssp. *kafiristanica, indica* spp. *indica*, *indica* ssp. *afghanica* and *indica* ssp. *chinensis*) species of *Cannabis.* This differentiation mainly reflects geographical differences, distinct chemical compositions and/or phenotypic variation of the *Cannabis* plants (McPartland, Clarke and Watson 2000; Hillig 2005; Linnaeus 1753).

*Cannabis* has been used by humans for different purposes for more than 5000 years. The fiber-type of *Cannabis* (hemp), where the major cannabinoid is cannabidiol (CBD), has been used as a source of fiber for textile production and oil seed. On the other hand, the drug-type of *Cannabis* (marijuana) have a higher content of the psychoactive compound Δ^9^-tetrahydrocannabinol (Δ^9^-THC) and for that reason has been used as a recreational drug but also in the treatment of pain and other medical conditions (for review see Russo 2011). Δ^9^-THC is widely used in pharmaceutical formulations for the treatment of several medical conditions. Several synthetic *Cannabis*-based preparations such as dronabinol (Marinol^®^, Unimed Pharmaceuticals, Inc, Marietta, GA, USA), nabilone (Cesamet^®^, Valeant Pharmaceuticals North America, Aliso Viejo, CA, USA), Δ^9^-THC and CBD (Sativex^®^, GW Pharmaceuticals plc, Histon Cambridge, United Kingdom) have been used in the USA, Canada and other countries as an authorized treatment for nausea and vomiting in cancer chemotherapy, appetite loss in acquired immune deficiency syndrome and symptomatic relief of neuropathic pain in multiple sclerosis. The number of applications for Δ^9^-THC, CBD and other cannabinoids is still increasing with more pharmaceutical applications being investigated (Carlini 2004; Pertwee 2009).

Cannabinoids are terpenophenolic compounds, produced from fatty acids and isoprenoids precursors as part of the secondary metabolism of *Cannabis*. The main cannabinoids produced by *Cannabis* are Δ^9^-tetrahydrocannabidiol (THC), CBD and cannabinol (CBN), followed by cannabigerol (CBG), cannabichromene (CBC) and other minor constituents (Flores-Sanchez and Verpoorte 2008). The biosynthesis of cannabinoids takes place mainly in the secretory head cells of the glandular trichomes, especially in the capitate-stalked glandular hairs (Happyana *et al.*^2013^). In the plant, Δ^9^-THC and CBD are involved in the defense response against pathogens (McPartland 1984), CBG and CBD are mildly antifungal (Elsohly *et al.*^2017^) and Δ^9^-THC is also involved in UV light protection (Russo 2011).

Phytocannabinoids are plant-based cannabinoids. Besides the genus *Cannabis*, there are other examples of plants that produce cannabinoid-like compounds. *Helichrysum umbraculigerum*, a flowering plant from Southern Africa, produces cannabigerol and cannabigerolic acid (CBGA) in its aerial parts (Bohlmann and Hoffmann 1979) and the moss *Radula marginata* produce perrottetinic acid (Toyota *et al.*^2002^). If not especially mentioned as *C. sativa* derived cannabinoids, structurally related compounds with a terpenophilic skeleton naturally synthesized by plants sources are also called phytocannabinoids. For a simplified designation, the term ‘cannabinoid’ will be used to refer to phytocannabinoids in this review.

Endocannabinoids are endogenous metabolites found in the members of the phylum Chordates, which bind to specific receptors of the endocannabinoid system (ECS). Recently, endocannabinoids were also discovered in algae, bryophytes and monilophytes (Gachet *et al.*^2017^). The main endocannabinoids are molecules derived from arachidonic acid, anandamide and 2-arachidonoylglycerol (Devane *et al.*^1992^; Mechoulam *et al.*^1995^; Sugiura *et al.*^1995^). These substances have a local effect and short life before being degraded by two well-characterized enzymes, the fatty acid amide hydrolase and monoacylglycerol lipase (Cravatt *et al.*^1996^; Dinh *et al.*^2002^). It is worth to note that synthetic compounds with no direct structural relation to plant cannabinoids have been designed in the past. These synthetic cannabinoids are chemicals developed to interact with the receptors of the ECS and are mostly known as illicit substances.

The ECS is a ubiquitous lipid signaling system with important homeostatic and physiological functions that include modulation of pain and inflammation. The name is derived from cannabinoids, because its first studies with cannabinoids led to the discovery and elucidation of the ECS and its biological functions. The isolation of Δ^9^-THC from *Cannabis* (Gaoni and Mechoulam 1964; Mechoulam 1970; Mechoulam and Gaoni 1965) led to the discovery and characterization of the specific mechanism of action of cannabinoids, by the identification of specific binding sites in the brain (Devane *et al.*^1988^; Herkenham *et al.*^1991^). This allowed the molecular cloning (Matsuda *et al.*^1990^) of cannabinoid CB_1_ receptor (CB_1_) as main cellular target. Later, a second peripheral receptor CB_2_ was identified (Munro, Thomas and Abu-Shaar 1993). Endocannabinoids are natural metabolites that stimulate this receptor type. Δ^9^-THC is a potent activator of the CB_1_ receptor, while the non-psychoactive CBD is a very low-affinity CB_1_ ligand. Despite this fact, CBD modulate the effect of Δ9-THC via direct blockade of CB_1_ receptor (McPartland *et al.*^2015^). This modulation attenuates some of the side effects of Δ^9^-THC such as anxiety, dysphoria, panic reactions and paranoia, and is also known to improve the Δ^9^-THC therapeutic activity (Izzo *et al.*^2009^; Russo 2011).

The global legislation for the use of medical *Cannabis* changes rapidly, and currently *Cannabis* is legal as therapeutic agent in 23 states of the United States as well as in the Netherlands, Germany, Czech Republic, Canada and Israel. There are still several drawbacks in the production of medical THC and other cannabinoids, especially related to the legal regulations for the cultivation of *Cannabis* in most countries. Furthermore, chemical synthesis of cannabinoids has failed to be a cost-effective alternative mainly because of complex synthesis leading to high production cost and low yields. A new alternative is the use of a biotechnology-based synthetic biology approach for a cost-effective, environmentally friendly, high-quality and reliable source of cannabinoids.

Agricultural production of cannabinoids faces several challenges such as plant susceptibility to climate and diseases, no GAP standardization, low content of less-abundant cannabinoids, need for extraction of cannabinoids by chemical processing and legal and social factors related to the potential for illicit use of the plant. Currently, Δ^9^-THC and CBD used as therapeutic agents are either extracted from the plant or chemically synthesized. Biosynthesis of cannabinoids by engineered microbial strains could be an alternative strategy for the production of cannabinoids.

The identification of the enzymes involved in the cannabinoids biosynthetic pathway enables the reconstruction of the pathway using a suitable heterologous host system. A synthetic biology approach can be especially interesting for the production of less-abundant cannabinoids, but also work as a platform for the discovery and testing of unknown enzymes responsible for the biosynthesis of various rare cannabinoids and derivatives thereof. Microbial production can provide a competitive and efficient way for easy and high yield biosynthesis of rare cannabinoids. Some examples of such compounds are tetrahydrocannabivarin, cannabigerol and cannabichromene that are cannabinoids with therapeutic interest, which are difficult to obtain (Appendino *et al.*^2008^; Bolognini *et al.*^2010^; Davis and Hatoum 1983; Elsohly *et al.*^2017^; Hill *et al.*^2010^; Ligresti 2006; Wilkinson *et al.*^2007^).

Recently, important developments in the microbial biosynthesis of cannabinoids were achieved. The expression of THCA synthase in the yeast *Komagataella phaffii* allowed the bioconversion of CBGA to Δ9-tetrahydrocannabinolic acid (Δ^9^-THCA) (Zirpel, Stehle and Kayser 2015). In addition, a patent application was filed, referring the microbial biosynthesis of cannabinoids in genetically engineered microorganisms (Poulos and Farnia 2016). In this review, we will explore synthetic biology as an alternative approach for the biosynthesis of cannabinoids in heterologous systems. We present microbial biosynthesis as a novel biotechnological solution for the production of pharmaceutical cannabinoids. The review will start with a brief overview of *Cannabis* phytochemistry focusing on the biosynthesis of cannabinoids. This will be followed by a comprehensive description of the key steps of the pathway, precursor needs and the desired features of the chassis organism.

## BIOSYNTHESIS IN *CANNABIS SATIVA*

The biosynthesis of cannabinoids starts with the short-chain fatty acid, hexanoic acid. Initially, the fatty acid is converted to its coenzyme A (CoA) form by the activity of an acyl activating enzyme (Stout *et al.*^2012^). Subsequently, olivetolic acid (OA) is biosynthesized by the action of a type III polyketide synthase (PKS) and a polyketide cyclase (olivetolic acid cyclase [OAC]). The PKS olivetol synthase (OLS) converts one molecule of hexanoyl-CoA and three molecules of malonyl-CoA to olivetol followed by the C2-C7 aldol cyclization to OA by the OAC (Gagne *et al.*^2012^; Raharjo *et al.*^2004^). A geranyl diphosphate:olivetolate geranyltransferase, named cannabigerolic acid synthase (CBGAS), is responsible for the C-alkylation by geranyl diphosphate (GPP) to CBGA (Fellermeier and Zenk 1998). Finally, three different oxidocyclase enzymes catalyze the oxidative cyclization of the monoterpene moiety of CBGA for the biosynthesis of Δ^9^-THCA, cannabidiolic acid (CBDA) and cannabichromenic acid (CBCA) (Morimoto *et al.*^1998^; Sirikantaramas *et al.*^2004^; Taura *et al.*2007b). The enzymes involved in cannabinoids biosynthesis in *C. sativa* L. are summarized in Table 1 and the biosynthetic pathway is described in Fig. 2. Neutral form of these cannabinoids is the result of a non-enzymatic decarboxylation that usually happens during the plant material storage, by heat (smoking or baking) or sunlight exposure (de Meijer *et al.*^2003^).

**Table 1.** List of the enzymes involved for the biosynthesis of cannabinoids in *C. sativa* L.

Enzyme	Abbreviations	Accession no.^a^	EC no.	References
Acyl activating enzyme 1	AAE1	AFD33345.1	6.2.1.1	(Stout *et al.*^2012^)
Olivetol synthase	OLS	AB164375	2.3.1.206	(Taura *et al.*^2009^)
Olivetolic acid cyclase	OAC	AFN42527.1	4.4.1.26	(Gagne *et al.*^2012^)
Cannabigerolic acid synthase	CBGAS	US8884100B2^b^	2.5.1.102	(Fellermeier and Zenk 1998)
				(Page and Boubakir 2012)
Tetrahydrocannabinolic acid synthase	THCAS	AB057805	1.21.3.7	(Sirikantaramas *et al.*^2004^)
Cannabidiolic acid synthase	CBDAS	AB292682	1.21.3.8	(Taura *et al*. 2007b)
Cannabichromenic acid synthase	CBCAS	WO 2015/196275 A1^c^	1.3.3-	(Morimoto *et al.*^1998^)
				(Page and Stout 2015)

a Genbank

b Patent number

c application number

### Polyketide pathway for the biosynthesis of olivetolic acid and biosynthesis of cannabinoids terpene precursor geranyl diphosphate

The synthesis of OA starts with hexanoic acid. In the *Cannabis* plant, the origin of this fatty acid in the trichomes has not yet been elucidated. The formation of hexanoic acid by a *de novo* biosynthesis route is suggested by data showing high expression of an acyl carrier protein (ACP) and a 3-keto-ACP reductase enzyme in the glandular trichomes in comparison with plant leaves. According to this hypothesis, hexanoic acid would be synthesized by an early termination of the fatty acid biosynthesis and the action of a specific acyl-ACP thioesterase (Marks *et al.*^2009^). Notwithstanding, it is also hypothesized that hexanoic acid might be derived from the lipoxygenase pathway through the degradation of C18 unsaturated fatty acids (Marks *et al.*^2009^; Stout *et al.*^2012^).

The hexanoyl-CoA synthetase 1 isolated from the transcriptome of glandular trichomes is most likely the enzyme responsible for the formation of hexanoyl-CoA for the cannabinoid pathway. Several findings support this hypothesis. This enzyme is specifically expressed in trichomes and is localized in the cytosol, as also suggested for OLS and OAC (Gagne *et al.*^2012^; Stout *et al.*^2012^; Taura *et al.*^2009^).

Olivetol and OA are classified as resorcinolic lipids (alkylresorcinol, resorcinolic acid) and are both biosynthesized via a polyketide pathway. The polyketide synthase OLS catalyzes the aldol condensation of hexanoyl-CoA with three malonyl-CoA units towards olivetol and the α-pyrones pentyl diacetic lactone and hexanoyl triacetic acid lactone. Recent reports show that OLS only produces OA in combination with the cyclase enzyme OAC (Fellermeier *et al.*^2001^; Gagne *et al.*^2012^; Raharjo *et al.*^2004^; Taguchi *et al.*^2008^; Taura *et al.*^2009^; Yang *et al.*^2016^). The OAC catalyzes the C2-C7 intramolecular aldol cyclization to OA by preserving the carboxylate moiety (Gagne *et al.*^2012^; Raharjo *et al.*^2004^).

The terpenoid part of the cannabinoid can be derived from two different precursor pathways, namely the mevalonate pathway (MVA) localized in the cytosol and the plastid localized non-MVA, also termed as 2-C-methyl-d-erythritol 4-phosphate or 1-deoxy-d-xylulose 5-phosphate pathway (MEP/DOXP pathway). In higher plants, the MVA pathway is mainly involved with the plant primary metabolism, whereas MEP pathway is the main contributor for secondary metabolism, including terpene production (in detail elsewhere, e.g. Eisenreich *et al.*^2004^; Hunter 2007). The MVA and MEP pathways are both responsible for the production of isopentenyl diphosphate (IPP) and dimethylallyl diphosphate (DMAPP), the two early precursors of terpenes. The condensation of IPP and DMAPP to produce GPP is catalyzed by the enzyme geranyl diphosphate synthase (Burke, Wildung and Croteau 1999). According to the information currently available, the most likely source of GPP for the production of the monoterpene moiety of cannabinoids in the plant is the MEP/DOXP pathway.

### Cannabinoids biosynthesis

The central precursor for cannabinoid biosynthesis, CBGA, is synthesized by the aromatic prenyltransferase CBGAS (Fellermeier and Zenk 1998; Page and Boubakir 2012) by the condensation of GPP and OA. CBGAS is mainly expressed in glandular trichomes of female flowers and young leaves of *Cannabis*, and the coding sequence could be functionally expressed in *Saccharomyces cerevisiae* cells, verifying the CBGAS activity in the microsomal fractions (Page and Boubakir 2012). Subsequently, the monoterpene moiety of CBGA is stereoselectively cyclized by the three different enzymes cannabichromenic acid synthase (Page and Stout 2015), cannabidiolic acid synthase (CBDAS) and tetrahydrocannabinolic acid synthase (THCAS). The CBDAS and THCAS belong to the berberine bridge enzyme family, oxidoreductases with a covalently bound flavin adenine dinucleotide (FAD) (Kutchan and Dittrich 1995). Both enzymes use molecular oxygen for the regeneration of the FAD cofactor, releasing equimolar amounts of hydrogen peroxide and a particular product (Sirikantaramas *et al.*^2004^; Taura *et al.*2007a). The THCAS is well characterized, and a crystal structure is available (Shoyama *et al.*^2012^). The enzyme possesses a signal peptide for the secretory pathway, one disulfide bond and eight possible Asn glycosylation sites. Additionally, the enzyme was already functionally expressed in *Spodoptera frugiperda* (Sf9) insect cells (Sirikantaramas *et al.*^2004^), *K. phaffii* (Taura *et al.*2007b; Zirpel, Stehle and Kayser 2015) and *S. cerevisiae* (Zirpel, Stehle and Kayser 2015).

## HETEROLOGOUS SYSTEMS FOR THE BIOSYNTHESIS OF CANNABINOIDS

### Special requirements of the host organism

Choosing a suitable host organism for production of a heterologous metabolite needs some general considerations, regarding the tools available for the expression of heterologous proteins (strains, vectors, promoters and signal peptides), genetic information and availability of classical genetic approaches as well as the accessibility of modern molecular biological tools. Other specific requirements would relate more to the specific pathway that needs to be introduced. Such factors could be the suitability of the host organism for production of particular precursors or cofactors as well as suitability of the host to express the necessary types of pathway enzymes. In Table 2, we compare a selection of potential microbial production hosts and evaluate their suitability within different categories. In the case of the cannabinoid biosynthesis, both precursors GPP and hexanoic acid should be provided in sufficient amounts by the chassis organism. The isoprenoid production has already been extensively studied and optimized for the production of artemisinin in *Escherichia coli* and *S. cerevisiae*, favoring the yeast as better platform organism (Paddon and Keasling 2014). Nevertheless, early stage optimizations of isoprenoid production are also documented for the oleaginous yeast *Yarrowia lipolytica* (Sharpe, Ye and Zhu 2014) and the methylotrophic yeast *K. phaffii* (Liu *et al.*^2014^).

**Table 2.** Comparison of different microbial expression hosts regarding their capacity of heterologous cannabinoid biosynthesis.

	Genetic tools available	Strains, promoters, vectors	Plant protein expression capacity	Posttranslational modifications	GPP engineering	Hexanoic acid engineering	Acetyl-CoA pool engineering
*Escherichia coli*	+++	+++	+	–	++	+	+
*Saccharomyces cerevisiae*	+++	+++	++	++	+++	++	+++
*Komagataella phaffii (Pichia pastoris)*	+	++	+++	++	+		
*Kluyveromyces marxianus*	++	+	++	++		++	
*Yarrowia lipolytica*	+	+	++	++	+	++	

+++, many publications available, well established; ++, publications available, optimization potential; +, first publications available, not yet established/not working; –, not possible; ‘empty’, not yet described.

A clear challenge in heterologous production of cannabinoids is the production of hexanoic acid. The most efficient microbial hexanoic acid formation reported to date is by Cheon *et al.* (2014), who described the production of up to 142 mgL^−1^ of hexanoic acid in *Kluyveromyces marxianus* using a pathway that may be transferrable to other yeasts.

Looking at the characteristics of the enzymes of the late cannabinoid pathway, a prokaryotic host seems not to be feasible. CBGAS is an integral membrane protein, making high titer of functional expressed protein in *E. coli* rather unlikely. In addition, the FAD-dependent oxygenases THCAS and CBDAS possess a disulfide bond and several *N*-glycosylation sites excluding thereby the use of prokaryotic hosts (Shoyama *et al.*^2012^; Zirpel, Stehle and Kayser 2015).

All precursors needed for the cannabinoid biosynthesis, GPP, malonyl-CoA and hexanoyl-CoA, are derived from acetyl-CoA. Therefore, a strategy to boost the acetyl-CoA pool is essential to reach high yields. Recently, *S. cerevisiae* was used to rewire the central carbon metabolism (Meadows *et al.*^2016^). By the addition of four genes only, the engineered yeast is able to produce high amounts of cytosolic acetyl-CoA accompanied with an improved pathway redox balance, a reduced ATP requirement and CO_2_ loss. Since *S. cerevisiae* also is one of the most developed host organisms in respect to knowledge and molecular biology tools, this organism therefore is a promising chassis organism for the heterologous biosynthesis of cannabinoids. In the following, we will therefore focus on the establishment of *S. cerevisiae* as a platform organism for the heterologous biosynthesis of cannabinoids. A serious alternative, however, would be *K. phaffii*, which is well known for secreted heterologous synthesized enzymes. Zirpel, Stehle and Kayser (2015) showed that this yeast is also able to produce high levels of intracellular accumulated THCAS.

### Metabolic engineering of *Saccharomyces cerevisiae* to produce cannabinoids

In order to rewire the *Saccharomyces cerevisiae* metabolism for the biosynthesis of cannabinoids, a combined approach comprising the adaptation of native metabolic pathways, the assembly of heterologous metabolic pathways and protein engineering is needed to obtain a cell factory that produces cost-effective cannabinoids. Looking at the biosynthetic pathway of THCA in *C. sativa*, the pathway can be divided into three parts: (i) GPP supply, (ii) synthesis of OA and (iii) the actual cannabinoid formation.

### GPP supply

**Figure 1.**
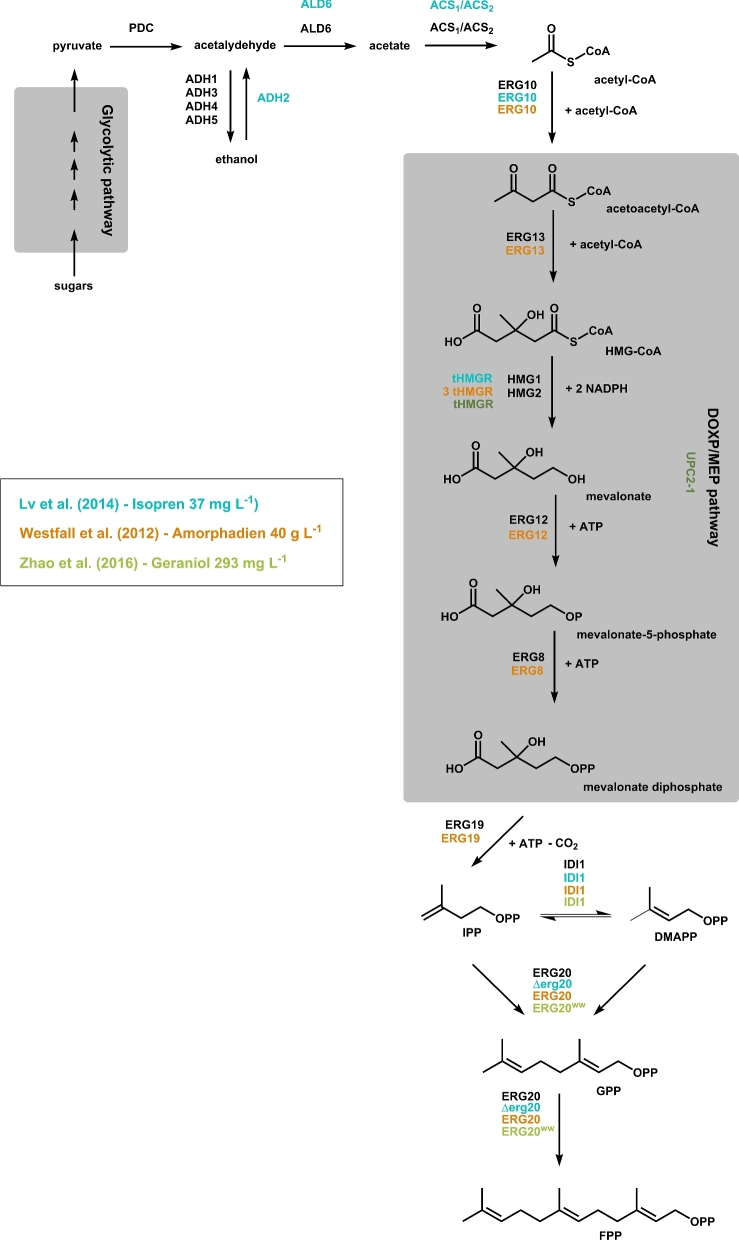
Isoprenoid formation in *S. cerevisiae*. The isoprenoid biosynthesis starts with acetyl-CoA, which is derived from the glycolytic pathway. At the end of the MVA, the both isoprenoids IPP and DMAPP are formed. Subsequently, GPP and FPP are formed by the ERG20 protein. The different colors represent the different strategies applied for improved isoprenoid production. HMG-CoA—3-hydroxy-3-methylglutaryl coenzyme A, IPP—isopentenyl diphosphate, DMAPP—dimethylallyl diphosphate, GPP—geranyl diphosphate, FPP—farnesyl diphosphate, PDC—pyruvate decarboxylase, ADH1-5—alcohol dehydrogenase, ALD6—aldehyde dehydrogenase, ACS_1_/ACS_2_—acetyl-coA synthetase, ERG10—acetyl-CoA C-acetyltransferase, ERG13—3-hydroxy-3-methylglutaryl-CoA synthase, tHMGR—truncated 3-hydroxy-3-methylglutaryl-CoA reductase, ERG12—mevalonate kinase, ERG8—phosphomevalonate kinase, ERG19—mevalonate diphosphate decarboxylase, IDI1—isopentenyl diphosphate:dimethylallyl diphosphate isomerase, ERG20—farnesyl diphosphate synthetase, ERG20^WW^—ERG20-F96W-N127W, Δerg20—ERG20 knock out, UPC2-1—sterol regulatory element binding protein.

*Saccharomyces cerevisiae* serves as a model and excellent organism for heterologous isoprenoid production (Nevoigt 2008), and several promising metabolic engineering strategies were identified by *in silico* profiling (Gruchattka *et al.*^2013^). Up to date, many strategies were tested to improve the isoprenoid production in yeast, whereas most are focusing on MVA pathway engineering (Fig. 1). In most cases, a truncated version of 3-hydroxy-3-methylglutaryl CoA reductase (HMGR) is overexpressed since HMGR was identified as the rate-limiting enzyme of the MVA pathway (Ohto *et al.*^2009^). Recently, the co-overexpression of all MVA pathway genes resulted in the production of 40 gL^−1^ amorphadiene, a precursor of the antimalarial agent artemisinin (Westfall *et al.*^2012^). Lv *et al.* (2014) optimized additionally the native acetyl-CoA pathway. The co-overexpression of pyruvate decarboxylase, alcohol dehydrogenase 3, aldehyde dehydrogenase and the both acetyl-coA synthetases resulted in an enhanced isoprene biosynthesis and might be an option to improve isoprenoid biosynthesis further.

In contrast to plants, yeasts usually do not carry a specific GPP synthase (GPPS). In yeast, the farnesyldiphosphate synthase (FPPS; ERG20) possesses a GPPS activity, while GPP occurs exclusively as an intermediate of farnesyl diphosphate synthesis. The highest yields so far were obtained with the production of sesquiterpenes or even larger terpenes like miltiradiene (Zhou *et al.*^2012^) or artemisinin (Paddon *et al.*^2013^). Less effort was made on the optimization in monoterpene production and the overall yields reported are lower. However, for cannabinoid production in yeast a high production rate of GPP is necessary. Saturated mutagenesis of Lys197 residue of the ERG20 protein resulted in six strains (K197G, C, S, T, D, E) with an improved monoterpenol production but also with some growth impairment (Fischer *et al.*^2011^). Even better results were obtained by engineering the ERG20 protein into a geranyl diphosphate synthase (ERG20-F96W-N127W = ERG20^WW^) (Ignea *et al.*^2014^). The introduction of a larger side chain in position 96 (F96W) blocks the part of the active site, thereby hindering the FPP synthase activity without affecting the synthesis of GPP. ERG20 is a homodimeric protein whereas the N127 residue of one subunit is part of the active site of the other subunit (Fernandez, Kellogg and Poulter 2000; Ignea *et al.*^2014^). The replacement of the N127 residue by a tryptophan results in an abolished FPP synthesis, but the GPP activity remains. The overexpression of the N127W variant in a wild-type yeast strain leads to the formation of heterodimeric ERG20 proteins (endogenous ERG20/ERG20^WW^) reducing thereby the overall FPP synthase activity and eliminates the need for mutation of the endogenous *ERG20* gene to reduce the FPP pool (Ignea *et al.*^2014^). Finally, the co-overexpression of IDI1, tHMG1 and UPC2-1 together with the ERG20^WW^ protein resulted in a significantly improved geraniol production (Zhao *et al.*^2016^). A similar approach could be adapted to improve the GPP supply for heterologous cannabinoid biosynthesis in *S. cerevisiae*.

### Olivetolic acid synthesis

*Saccharomyces cerevisiae* does not typically metabolize fatty acids as substrate, therefore its production relies on endogenous biosynthesis. The fatty acid composition in *S. cerevisiae* is rather simple, consisting mostly of C16 and C18 fatty acids (Klug and Daum 2014; Oh and Martin 2006). Trace amounts of short-chain fatty acids (SCFA) or medium-chain fatty acids (MCFA) are naturally produced by *S. cerevisiae* and certain strains used in wine and sake fermentation are known to produce higher levels (Aritomi *et al.*^2004^; Patel and Shibamoto 2002). The biosynthesis of fatty acids requires several substrates and cofactors such as acetyl-CoA, ATP and NAD(P)H. The first committed step of fatty acid biosynthesis is the conversion of acetyl-CoA to malonyl-CoA. This reaction is performed by the enzyme acetyl-CoA carboxylase and its activity is highly regulated (Hablacher *et al.*^1993^; Woods *et al.*^1994^). *De novo* biosynthesis of fatty acid in yeast is carried out by the fatty acid synthase (FAS), a multifunction protein complex in which all steps of fatty acid synthesis are integrated. The yeast FAS is classified as a eukaryote type I and this enzyme complex is divided in 2 subunits (Fas1p and Fas2p), each exhibiting more than one enzymatic activity. Fas1p harbors acetyl transferase, enoyl ACP reductase, hydroxyl acyl ACP dehydratase and malonyl transferase activities; and Fas2p contains the ACP, ketoacyl reductase, ketoacyl synthase and phosphopantheteine transferase activities (Jenni *et al.*^2007^; Klug and Daum 2014; Tehlivets, Scheuringer and Kohlwein 2007).

Up to date, several strategies were tested to produce SCFA in yeast species, usually involving the introduction of heterologous enzymes. Several examples of the production of SCFA were demonstrated by the introduction of heterologous FAS. The introduction of *Homo sapiens* type I fatty acid synthase and specific short-chain thioesterase in *S. cerevisiae* increased *in vivo* octanoic acid and total SCFA production (Leber and Da Silva 2014). A similar strategy could be adapted for the production of hexanoic acid by testing different heterologous short-chain thioesterases. Many fungal secondary metabolites are fatty-acid-derived molecules biosynthesized by the interaction of dedicated FAS with a PKS. One of the most notorious examples of this type of secondary metabolites is the biosynthesis of aflatoxin (AF) and sterigmatocystin (ST) in some species of the filamentous fungus *Aspergillus*. Previous studies have shown that the synthesis of AF and ST begins with the assembly of a C6 fatty acid from acetyl-CoA and 2 units of malonyl-CoA catalyzed by a specialized FAS, hexanoate synthase (Hitchman 2001).

Recently, different studies demonstrated the production of extracellular SCFA and MCFA by the modification of the native FAS or construction of a synthetic FASs in *S. cerevisiae*. Rational engineering of the cytosolic FAS allowed the reprogramming of the chain-length control with successful biosynthesis of SCFA and MCFA (Gajewski *et al.*^2017^). In another study, the creation of a synthetic FASs by the integration of heterologous enzymes showed the capability to biosynthesize SCFA, MCFA and methyl ketones (Zhu *et al.*^2017^).

As mentioned before, the biosynthesis of OA starts with the conversion of the free fatty acid hexanoic acid to hexanoyl-CoA. In *Cannabis*, this step is done by an acyl-CoA synthetase with high specificity for hexanoic acid. *Saccharomyces cerevisiae* contains four fatty acyl-CoA synthetases (FAA) named FAA1, FAA2, FAA3 and FAA4. FAA1, FAA3 and FAA4 have preference for long-chain fatty acids (C12:0 to C18:0), in contrast with FAA2 that accepts a wide range of fatty acid chain lengths with a preference for medium chains (C9:0-C13:0) (Johnson *et al.*^1994^). Since *S. cerevisiae* has no specific acyl-CoA synthetase for SCFA, it may be beneficial to introduce a *Cannabis* hexanoyl-CoA synthetase or a homolog from other organism to achieve an efficient conversion of hexanoic acid to hexanoyl-CoA in yeast.

**Figure 2.**
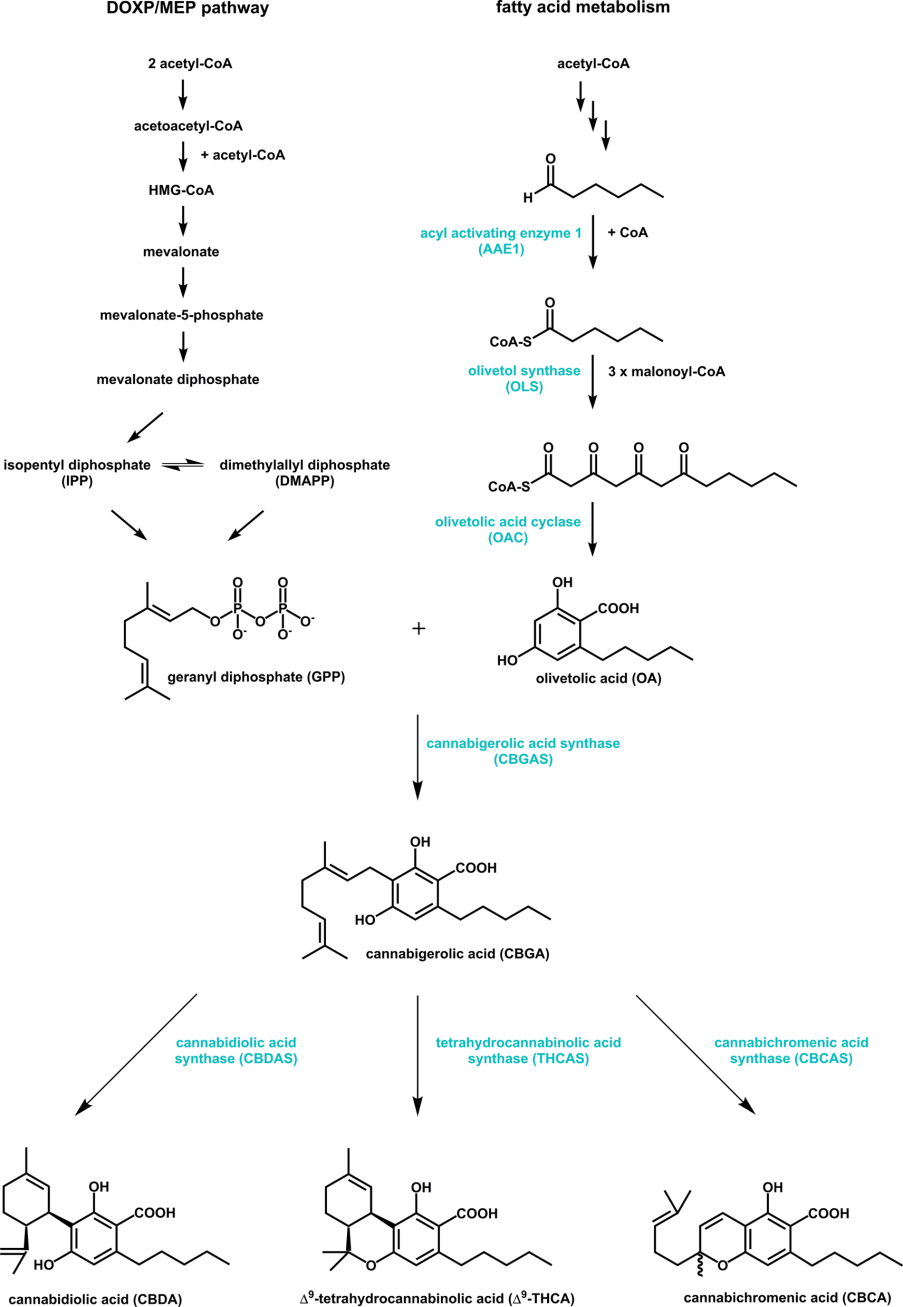
Biosynthetic pathway of cannabinoids in *C. sativa*. Highlighted enzymes have to be transferred into a heterologous host as *S. cerevisiae* exhibiting a mevalonate pathway.

The biosynthesis of OA from hexanoyl-CoA is performed by the combination of two cytosolic-located enzymes, OLS and OAC (Fig. 2). These two enzymes have been demonstrated to be active *in vivo* in *S. cerevisiae* with resulting formation of both olivetol and OA (Gagne *et al.*^2012^).

### Implementation of cannabinoid forming enzymes

With GPP and OA available in the production host, only one enzymatic step is necessary to obtain CBGA, which is the central intermediate of the cannabinoid pathway (Fig. 2). The native enzyme responsible for the prenylation of OA is an integral membrane enzyme of which functional overexpression has turned out to be challenging. Nevertheless, the native enzyme has been expressed in *Saccharomyces cerevisiae* cells, but the overall activity is low and a high side-product formation (5-geranyl-olivetolate) was observed (Page and Boubakir 2015). Besides the integral membrane prenyltransferases in plants, soluble prenyltransferases are known from fungi and bacteria. Kuzuyama, Noel and Richard (2005) published a crystal structure of a soluble prenyltransferase NphB from *Streptomyces* sp. strain CL190 that is specific for GPP as prenyl donor and exhibits a broad substrate specificity towards aromatic substrates. They were able to show that the enzyme accepts olivetol and OA as prenyl acceptor. Furthermore, it was shown that NphB prenylates olivetol in the C2 and C4 position (Kumano *et al.*^2008^). Thus, NphB represents a potential alternative to replace the native CBGAS in a biotechnological production of cannabinoids. Indeed, the co-expression of the *nphB* and *thca* coding sequences in *K. phaffii* resulted in the successful synthesis of THCA in enzyme extracts containing OA and GPP (unpublished results).

Once the expression of an OA prenylating enzyme is established in yeast, the addition of one respective enzyme leads to the formation of different cannabinoids like THCA, CBDA and CBCA. For the THCAS, a whole cell bioconversion is already established in *S. cerevisiae* and *K. phaffi* (Zirpel, Stehle and Kayser 2015). Yeast cells expressing *thcas* were able to produce up to 360 mgL^−1^ THCA after CBGA feeding, demonstrating the capacity of yeast in the biosynthesis of cannabinoids. As the sequence and expression for CBDAS is already described (Taura *et al.*2007a), the THCAS can be easily exchanged with a CBDAS. However, expression levels are lower than reported for THCAS (unpublished data).

### Diversified cannabinoids

The microbial production facilitates the possibility to design new cannabinoids with novel activities or improved pharmacokinetics through the implementation of tailoring enzymes. Cannabinoids are extremely hydrophobic compounds, for medical formulations cannabinoids are usually dissolved in oil or solvents, which may not be well tolerated by users (Scully 2007). The introduction of hydroxyl, carbonyl, carboxyl or glycosyl groups enhances the solubility of molecules in general. In 1980s, fungal and bacterial strains were already used to transform THC into polar derivatives (Binder and Meisenberg 1978; Binder and Popp 1980; Fukuda, Archer and Abbott 1977), as well as plant cell suspension cultures were used for the biotransformation of cannabinoids (Akhtar, Mustafa and Verpoorte 2015; Braemer and Paris 1987; Hartsel, Loh and Robertson 1983). Nevertheless, the overall yield of the metabolites was too low for pharmacological evaluation of the activity.

**Figure 3.**
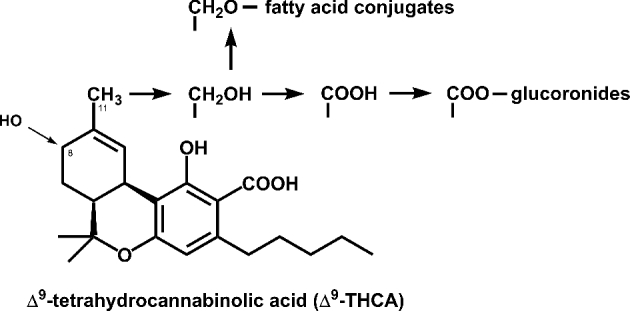
THCA metabolization products in humans. The C_11_ position is the major attacked site, but C_8_ position can be also hydroxylated (Grotenhermen 2003).

In humans, THC is metabolized in the liver mainly by microsomal hydroxylation and oxidation, but also allylic oxidation, epoxidation, aliphatic oxidation, decarboxylation and conjugation reactions were reported (Grotenhermen 2003). This leads to nearly 100 different identified metabolites for THC (Harvey and Brown 1991). THC is mainly hydroxylated on position C-11 by cytochrome P450 enzymes resulting in 11-hydroxy-tetrahydrocannabinol (11-OH-THC) and further oxidation leads to 11-nor-9-carboxy-tetrahydrocannabinol, the most important non-psychotropic metabolite (Fig. 3). Since 11-OH-THC seems to be three to seven times more potent than THC in animal tests (Karler and Turkanis 1987), and 11-OH-THC possesses still anti-inflammatory and analgesic properties (Burstein 1999), the elongation of the heterologous pathway by P450 enzymes might be a promising approach to produce THC derivatives. Glycosylation may also be an interesting strategy to alter the physicochemical properties. Plant glycosyltransferases (UGTs) are known for their relaxed substrate specificity (Bowles *et al.*^2006^; Hansen *et al.*^2009^). Recently, the UGT76G1 from *Stevia rebaudiana* was used to produce primary, secondary and tertiary glycosylations cannabinoid glycosides (Hardman, Brooke and Zipp 2017). The use of a second enzyme from *Oryza sativa* resulted in the transfer of a second glucose residue onto cannabinoid monoglycosides with a greatly improved water solubility.

The implementation of the cannabinoid biosynthesis in a heterologous host provides a platform where some of these tailoring enzymes can be functionally expressed. This enables the production of significant amounts of these derivatives and may be the key to unlock their pharmacological potential.

## OUTLOOK

The discovery and characterization of all key enzymes involved in the biosynthesis of the main cannabinoids Δ^9^-THC and CBD, allows for the production of these compounds by heterologous host organisms. The use of synthetic biology for the microbial biosynthesis of cannabinoids can revolutionize the production of medical cannabinoid drugs. Besides the main cannabinoids Δ^9^-THC and CBD, more than 100 cannabinoids compounds are described with little knowledge available regarding their producing pathways and potential applications. Synthetic biology could be used to create a chassis organism for the study and characterization of the enzymes involved in the biosynthesis of these less-abundant cannabinoids or derivatives thereof and allows the production of these compounds by a scalable fermentation process. This can have an enormous impact on the availability of rare cannabinoids to be tested in clinical trials to evaluate their efficacy as medical drugs. Furthermore, microbial production can support the design of novel cannabinoids with enhanced properties by the incorporation of tailoring enzymes. Together, these strategies will help to support the potential value of cannabinoids as pharmaceutical drugs.

***Conflict of interest.*** None declared.
